# Randomized phase III trial of concurrent chemoradiotherapy vs accelerated hyperfractionation radiotherapy in locally advanced head and neck cancer

**DOI:** 10.1093/jrr/rrt054

**Published:** 2013-06-05

**Authors:** Imjai Chitapanarux, Ekkasit Tharavichitkul, Pimkhuan Kamnerdsupaphon, Nantaka Pukanhapan, Roy Vongtama

**Affiliations:** 1 Division of Therapeutic Radiology and Oncology, Faculty of Medicine, Chiang Mai University, 110 Intawarorose Road, Chiang Mai, 50200, Thailand; 2 St Teresa Comprehensive Cancer Center, Stockton, California, Quail Lakes Dr, CA 95207, USA

**Keywords:** locoregionally advanced head and neck cancer, concurrent chemoradiotherapy, concomitant boost

## Abstract

The aim of this study was to compare the efficacy and safety of concurrent chemoradiotherapy (CCRT) vs accelerated hyperfractionation with concomitant boost (CCB) as a primary treatment for patients with Stage III*–*IV squamous cell carcinoma of head and neck (SCCHN). A total of 85 non-metastatic advanced SCCHN patients were accrued from January 2003 to December 2007. Of these, 48 and 37 patients received CCRT and CCB, respectively. The patients were randomized to receive either three cycles of carboplatin and 5-fluorouracil plus conventional radiotherapy (CCRT, 66 Gy in 6.5 weeks) or hybrid accelerated radiotherapy (CCB, 70 Gy in 6 weeks). The primary endpoint was determined by locoregional control rate. The secondary endpoints were overall survival and toxicity. With a median follow-up of 43 months (range, 3–102), the 5-year locoregional control rate was 69.6% in the CCRT arm vs 55.0% in the CCB arm *(P =* 0.184). The 5-year overall survival rate was marginally significantly different *(P* = 0.05): 76.1% in the CCRT arm vs 63.5% in the CCB arm. Radiotherapy treatment interruptions of more than three days were 60.4% and 40.5% in the CCRT arm and CCB arm, respectively. The median total treatment time was 55.5 days in the CCRT arm and 49 days in the CCB arm. The rate of Grade 3*–*4 acute mucositis was significantly higher in the CCB arm (67.6% vs 41.7%, *P* = 0.01), but no high grade hematologic toxicities were found in the CCB arm (27.2% vs 0%). CCRT has shown a trend of improving outcome over CCB irradiation in locoregionally advanced head and neck cancer.

## INTRODUCTION

Concurrent chemoradiotherapy (CCRT) has emerged as an accepted standard of care for locally advanced head and neck squamous cell cancer. At least three meta-analyses have been published which have demonstrated the benefit of CCRT over radiotherapy alone [1*–*4]. However, hematologic toxicity and renal toxicity of CCRT often result in a poor compliance rate. An alternative method of radiation delivery is altered fractionation. A recent meta-analysis by Bourhis *et al*. [5] showed the positive impact on overall survival (OS) of altered fractionation regimens, specifically hyperfractionation (HFX) and accelerated fractionation by concomitant boost regimen (CCB). RTOG 90-03 [6] also revealed that both HFX and CCB gave significantly better locoregional control (LRC) than conventional fractionation. We initiated a prospective randomized clinical trial to test whether or not a CCB regimen has the same treatment outcome as CCRT.

## MATERIALS AND METHODS

### Patient eligibility

Patients were eligible if they had: squamous cell carcinoma of all sites of head and neck (except nasopharynx, nasal cavity and paranasal sinus, and salivary gland), Stage III or IV disease according to the 2002 American Joint Committee on Cancer (AJCC) criteria [7], no distant metastases, ECOG performance status of 0*–*1, age 18*–*75 years, adequate laboratory organ system function and were previously untreated. The institutional ethics committee approved the protocol. Written informed consent was obtained from all patients before entering this study. This was a phase III prospective randomized trial. The patients were randomized to Arm CCB or Arm CCRT in a 1:1 ratio.

### Radiotherapy

In the CCB arm, the treatment regimen planned to deliver a total dose of 70 Gy in six weeks. Patients were given 40 Gy (2 Gy per day), five days per week. In the fifth week of radiotherapy, an off cord dose was given with 1.8 Gy per day for the first daily fraction and a CCB dose of 1.2 Gy per day as the second daily fraction. In the last two weeks, a boost volume (covering only the primary tumor site) was treated twice daily, 1.8 Gy per fraction in the morning and 1.2 Gy per fraction in the afternoon. The interval between the two daily fractions was at least 6 h. After 40 Gy, we did not perform a re-plan technique. In the CCRT arm, conventional fractionation was used (66 Gy at 2 Gy per fraction, five days per week, over a total of 6.5 weeks). The treatment volume for both arms was the same. A shrinking field technique was used with the primary field arrangement, which included two opposed lateral fields to treat the primary tumor and upper neck nodes, and low anterior neck fields (supraclavicular fields) were treated to cover the lower neck nodes. For lateral fields, in general, the anterior border was placed at a 2-cm margin beyond the primary tumor, the posterior border was placed behind the spinous process, the superior border was placed to cover a 2-cm margin above the tumor, and the inferior border was placed at the arytenoid cartilage. The spinal cord was blocked at a dose of 40 Gy. An electron beam with appropriate energy was used to give a dose to the posterior neck field bilaterally. The low anterior low-neck field was treated with conventional fractionation to a dose of 50 Gy in both arms. All patients were treated with a 6-MV linear accelerator. Neither 3D conformal radiotherapy nor intensity-modulated radiation therapy were used in this study.

### Chemotherapy

In the CCRT arm, the patients received three cycles of chemotherapy on Days 1, 22 and 43. The regimen followed the 94-01 French Head and Neck Oncology and Radiotherapy group [8] protocol consisting of carboplatin and 5-FU. Carboplatin was given as a daily dose of 70 mg/m^2^/day for four days via intravenous bolus. 5-FU was given as a dose of 600 mg/m^2^/day for four days via continuous intravenous infusion.

### Toxicity assessment

During treatment, the patients in both arms were examined for acute radiation (skin, and mucosa) and chemotherapy toxicity, and scored using NCI-CTC version 2.0 [9]. Complete blood count and renal function were also performed weekly in all patients. Chemotherapy was withheld for patients who had Grade > 2 acute hematologic toxicity (Hb 8 *–* <10 g/dl, WBC ≥2.0 – <3.0 × 10^9^/l, platelet count ≥50.0 *–* <75.0 × 10^9^/l) until recovery to Grade ≤1. Radiotherapy was withheld in the patients who had Grade ≥ 3 leucopenia (WBC ≥1.0 *–* <2.0 × 10^9^/l), thrombocytopenia (≥10.0 *–* <50.0 × 10^9^/l) until recovery to Grade ≤ 1. Radiotherapy was also withheld in the patients who had Grade ≥ 3 radiation dermatitis (confluent moist desquamation, ≥1.5 cm diameter, not confined to skin folds, pitting edema) until recovery to Grade ≤ 2 (moderate to brisk erythema or patchy moist desquamation, mostly confined to skin folds and creases; moderate edema). Patients who developed Grade ≥ 3 radiation mucositis (confluent ulcerations or pseudomembrane; bleeding with minor trauma) were put on break until recovery to Grade ≤ 2 (only patchy ulcerations or pseudomembranes). Late radiotherapy toxicity was prospectively graded using the RTOG/EORTC Late Radiation Morbidity Scoring Scheme [10]. Late side-effects were recorded only in patients who survived > 6 months from the beginning of radiotherapy.

### Statistical analysis

CCRT and accelerated radiotherapy have both been shown to improve the probability of tumor-control outcome independently. The present study was designed as a non-inferiority trial comparing CCB with CCRT. It is not expected, by design, that CCB would be more efficacious, but rather that compliance with CCB would be higher due to the lack of chemotherapy. This higher compliance rate could result in a comparable efficacy, especially off-trial. Efficacy was analyzed on an as-treated basis. In the as-treated analysis, only data from patients continuing randomized treatment were considered for analysis, based on the time from random assignment to death. We used an as-treated analysis as it can be argued to provide a more conservative approach for a non-inferiority assessment. Given the results of the GORTEC 94-01 study [8], the 5-year OS with chemoradiotherapy in the French trial was 22.4%. We expected a 5-year OS of 25% in both our CCRT and CCB groups. In the OS analysis, in order to determine with 80% power, using a one-sided type I error of 0.20, the non-inferiority at 5-year OS of the two treatment arms, at least 106 patients per group had to be observed. The primary endpoint was LRC. The secondary endpoints included OS, compliance with the treatment, and toxicities. Non-inferiority in this study, in terms of 5-year OS, was tested with a 10% margin. OS and LRC were calculated according to the Kaplan-Meier method, and compared using the log-rank test. OS was calculated from the date of the last day of treatment to the date of death from any cause. *P*-values were considered to be statistically significant at *P* < 0.05. The statistical analyses were performed using SPSS version 17 (SPSS Inc., Somers, NY, USA).

## RESULTS

**Fig. 1.**
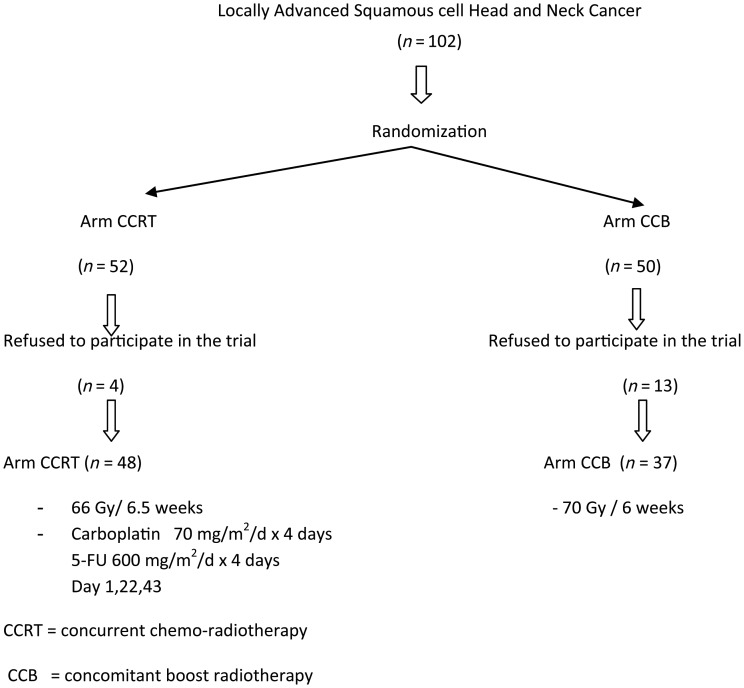
Study design of the trial. CCRT = concurrent chemoradiotherapy, CCB = concomitant boost radiotherapy.

Between January 2003 and December 2007, a total of 102 patients were enrolled and randomized. Due to poor accrual, the study was terminated and a final analysis was done in May 2012. Prior to the initiation of therapy, 17 patients refused to participate in the study and were referred to other centers near their residential area. Of the remaining 85 patients in the as-treated analysis, 48 patients received CCRT and 37 patients received CCB (Fig. 1). Baseline characteristics are shown in Table 1, and were balanced in both groups, except for age.

**Table 1.** Baseline characteristics

Characteristics	Arm CCRT (*n* = 48)	Arm CCB (*n* = 37)	*P*-value
Age (years)			
Median (range)	58.5 (28*–*70)	63.5 (40*–*77)	0.05
Sex			
Male/Female	38/10	26/11	0.19
AJCC Stage			
III (%)	22 (45.8)	13 (35.1)	0.32
IV (%)	26 (54.2)	24 (64.9)	
Primary tumor			
Oral cavity (%)	13 (27)	13 (35)	0.70
Oropharynx (%)	8 (17)	6 (16)	
Larynx (%)	25 (52)	16 (44)	
Hypopharynx (%)	2 (4)	2 (5)	

CCRT = concurrent chemoradiotherapy, CCB = concomitant boost.

### Treatment compliance

The median overall treatment time was 55.5 days (range, 46*–*103) in the CCRT arm, which was above our ideal time course of 46 days. In the CCB group, the median overall treatment time was 49 days (range, 42*–*69), which was more than our ideal plan of 42 days. Radiation interruption of >3 days due to acute toxicities was significantly greater in the CCRT arm (29 patients; 60.4%) vs the CCB arm (15 patients; 40.5%). Some patients had two or more acute side-effects concurrently at the time of treatment interruption. In the CCRT arm, the most common causes for interruption were Grade 3 mucositis (41.7%), followed by severe hematologic toxicity (27.2%), and skin toxicity (25%), whereas in the CCB arm, mucositis was the major cause of radiotherapy interruption (67.6%), followed by skin toxicity (16.2%). Also, the median duration of the radiotherapy treatment break was longer in the CCRT arm than in the CCB arm: 8 days (range, 4*–*18 days) vs 5 days (range, 4*–*13 days). Among the 48 patients in the CCRT arm, 30 patients (62.5%) received three cycles of chemotherapy and 18 patients (37.5%) received only two cycles, because initiation of the third cycle would have begun after radiation was already complete. No patients received only one cycle. Of the 48 patients, 25 (52.1%) had delayed cycles due to hematologic toxicity. Treatment compliance is shown in Table 2.

**Table 2.** Treatment compliance

Characteristics	Arm CCRT (*n* = 48)	Arm CCB (*n* = 37)
Chemotherapy compliance		
Delayed cycles (%)	25 (52.1)	
Omission cycles (%)	18 (37.5)	
Radiotherapy compliance (%) (treatment interruptions > 3 days)	29 (60.4)	15 (40.5)
Median (range) for overall treatment time (days)	55.5 (46–103)	49 (42–69)

CCRT = concurrent chemoradiotherapy, CCB = concomitant boost.

### Acute toxicities

No patient died of acute toxicity. The most common radiation side-effect amongst the patients was radiation mucositis. The rate of Grade 3–4 acute radiation mucositis was significantly higher in the CCB arm (67.6%) than in the CCRT arm (41.7%), *P* = 0.01. Grade 3*–*4 radiation skin toxicity did not differ between the two groups (16.2% in CCB group vs 25% in CCRT group, *P* = 0.33). As expected, severe hematologic toxicities were more frequently found in the CCRT arm: Grade 3*–*4 anemia, leucopenia and thrombocytopenia were found in 2.1%, 18.8% and 6.3%, respectively, in the CCRT arm, whereas no Grade 3*–*4 hematologic toxicities were found in the CCB arm. Table 3 shows the acute toxicities for both groups.

**Table 3.** Acute toxicities (scored by NCI-CTC version 2.0)

Toxicities	Arm CCRT (%) (*n* = 48)	Arm CCB (%) (*n* = 37)	*P*-value
Skin toxicity			
Grade 0–1	14 (29)	7 (18.9)	0.22
Grade 2	22 (46)	24 (64.9)	
Grade 3	12 (25)	6 (16.2)	
Grade 4	0	0	
Mucositis			
Grade 0–1	12 (25)	1 (2.7)	0.01
Grade 2	16 (33.3)	11 (29.7)	
Grade 3	20 (41.7)	25 (67.6)	
Grade 4	0	0	
Hemoglobin			
Grade 0–1	40 (83.3)	35 (94.6)	0.25
Grade 2	7 (14.6)	2 (5.4)	
Grade 3	1 (2.1)	0	
Grade 4	0	0	
Leukocyte			
Grade 0–1	0	33 (89.2)	0.00
Grade 2	39 (81.2)	4 (10.8)	
Grade 3	8 (16.7)	0	
Grade 4	1 (2.1 )	0	
Platelet			
Grade 0–1	44 (91.6)	36 ( 97.3)	0.30
Grade 2	1 (2.1)	1 ( 2.7)	
Grade 3	3 (6.3)	0	
Grade 4	0	0	
Renal toxicity			
Grade 0–1	43 (89.6)	35 (94.6)	0.34
Grade 2	5 (10.4)	2 (5.4)	
Grade 3	0	0	
Grade 4	0	0	

CCRT = concurrent chemoradiotherapy, CCB = concomitant boost.

### Late radiation toxicities

Late toxicities were assessed in 71 patients who survived > 6 months from the beginning of radiotherapy (46 patients for the CCRT arm, and 25 patients for the CCB arm). No Grade 4 toxicities were found in either arm. The most common late toxicity in both arms was Grade 2 mucositis (71.7%, 33/46 patients in the CCRT arm, and 76%, 19/25 patients, in the CCB arm). However, severe late radiation mucositis (Grade 3) was found more frequently in the CCB arm (8% vs 2.2%). Rates of late skin toxicity, late subcutaneous tissue fibrosis and late xerostomia did not differ between the two arms (Table 4).

**Table 4.** Late radiation toxicities (scored by RTOG/EORTC Late Radiation Morbidity Scoring Scheme)

Toxicities	Arm CCRT (%) (*n* = 46)	Arm CCB (%) (*n* = 25)	*P*-value
Skin			
Grade 1	11 (23.9)	6 (24)	**0.97**
Grade 2	32 (69.6)	17 (68)	
Grade 3	3 (6.5)	2 (8)	
Grade 4	0	0	
Subcutaneous			
Grade 1	34 (73.9)	10 (40)	**0.01**
Grade 2	12 (26.1)	14 (56)	
Grade 3	0	1 (4)	
Grade 4	0	0	
Mucous membrane			
Grade 1	12 (26.1)	4 (16)	**0.35**
Grade 2	33 (71.7)	19 (76)	
Grade 3	1 (2.2)	2 (8)	
Grade 4	0	0	
Salivary glands			
Grade 1	21 (45.7)	10 (40)	**0.89**
Grade 2	23 (50)	14 (56)	
SGrade 3	2 (4.3)	1 (4)	
Grade 4	0	0	

CCRT = concurrent chemoradiotherapy, CCB = concomitant boost.

### Treatment outcome

**Fig. 2.**
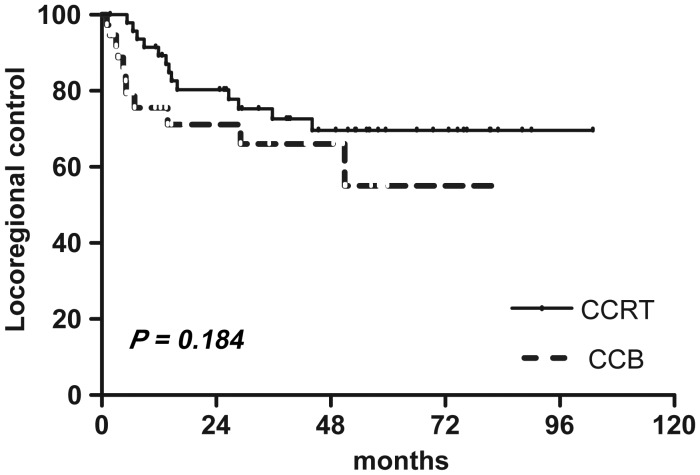
Locoregional control rates between the two groups.

**Fig. 3.**
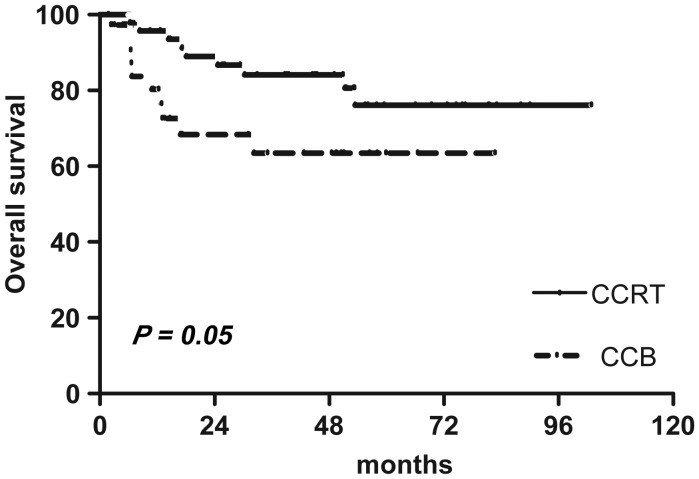
Overall survival rates between the two groups.

With a median follow-up of 43 months (range, 3*–*102), CCRT did not demonstrate a statistical difference (*P* = 0.18) in LRC compared with CCB radiotherapy: 69.6% (95% CI = 53.1, 81.2) in the CCRT arm vs 55.0% (95% CI = 28.1, 75.5) in the CCB arm (Fig. 2). However, the 5-year OS rate approaching was significantly greater *(P =*0.05) for the CCRT arm compared with the CCB arm, 76.1% (95% CI = 57.8, 87.3) vs 63.5% (95% CI = 42.0, 78.8), respectively. Kaplan-Meier curves for OS are shown in Fig. 3.

## DISCUSSION

CCRT is a standard of care for the curative treatment of locoregional, advanced squamous cell carcinoma of head and neck (SCCHN) [2, 11]. The survival benefit for CCRT compared with radiotherapy alone has been demonstrated in several meta-analyses [3*–*4]. Another treatment strategy that has overcome the poor results of conventional radiotherapy in this group of patients is altered fractionation, especially hyperfractionation and accelerated radiotherapy [12*–*13]. MARCH (Meta-analysis of Radiotherapy in Carcinoma of Head and Neck), a unique updated database of more than 6500 SCCHN patients, confirmed a survival benefit using altered fractionation. Additionally in the MARCH analysis, altered fractionated demonstrated a reduction in local failure, 23% at five years, as well as a benefit in LRC (13% reduction in the risk at five years) [5].

In the French trials, evaluating CCRT, carboplatin/5-FU demonstrated a significant improvement in LRC, disease-free survival, and OS at three and five years [8, 14]. Due to the significant number of elderly patients at our center, we also chose carboplatin instead of cisplatin because of the lower incidences of nephrotoxicity. Toxicity-wise, the CCRT arm demonstrated a similar acute toxicity profile to that of previous studies [15*–*22]. Most randomized trials of CCRT have shown Grade 3*–*4 mucositis ranging from 28*–*77% [15*–*20]. Our study found Grade 3*–*4 mucositis in 25% of patients.

In the altered fractionation arm, we selected CCB irradiation over hyperfractionation due to the busy workload of our center. In our study, no Grade 4 mucositis was found, but the rate of Grade 3 acute mucositis for patients was 67.6%. This was not surprising, as in previous studies looking at CCB irradiation the rate of Grade 3*–*4 confluent acute mucositis was quite high. Mak *et al*., in a study of CCB irradiation for base of tongue cancer, demonstrated a Grade 3*–*4 acute mucositis rate of 94% [23]. Comparing the two arms, we found a much higher frequency of Grade 3 mucositis in the CCB arm (67.6% CCB vs 41.7% CCRT). As expected, a higher incidence of Grade 3*–*4 acute hematologic toxicities (27.2%) was seen in the CCRT arm, whereas none were seen in the CCB arm. Hematologic toxicities were the main cause of delay of radiation treatment, prolonged overall treatment time, and also the poor compliance seen with the chemotherapy protocol. As seen in Table 2, 60.4% of patients in the CCRT arm had radiation treatment interruption of > 3 days, much greater than in the CCB arm (40.5%). To all the Grade 3*–*4 neutropenic patients, we administered GCSF and oral antibiotics upon diagnosis to mitigate a protracted overall treatment time. In our study population, 62.5% received all three planned cycles of chemotherapy, comparing favorably with 57% in the Canadian trial [24], and 65% in the French trial [14].

We did not find a significant difference in LRC between the two arms. The 5-year LRC rate for the CCRT arm was 69.6% (95% CI = 53.1, 81.2), comparing favorably with the 3-year LRC rate for the 94-01 French trial was 66% (95% CI = 51, 78) [14]. The 3-year OS rate for the French trial was 51% (95% CI = 39, 65), whereas our study had a higher 5-year OS rate of 76.1% (95% CI = 57.8, 87.3). In comparison with our CCB outcomes, Mak *et al*. at the MD Anderson Cancer Center [23] reported a 5-year LRC rate and an OS rate of 76% and 59%, respectively. In our study, the patients in the CCB arm had a slightly lower 5-year LRC rate, compared with Mak *et al*.: 55% (95% CI = 28.1, 75.5), but compared favorably in OS rate: 63.5%; (95% CI = 42.0, 78.8). However, comparisons between outcomes of the two studies should be tempered with the knowledge that our study had a mixed group of primary tumor sites, and nearly two-third of the patients in the CCB arm were graded Stage IV, whereas in the Mak *et al*. study all tumors were base of tongue at a range of stages, including a large percentage of node-negative and early-stage tumors.

Kader *et al*. retrospectively reported the outcomes for 321 patients with locally advanced oropharyngeal cancer treated with radiochemotherapy (RT-CT), accelerated fractionation radiotherapy (AccRT), or hypofractionated radiotherapy (HypoRT) (*n* = 157, 57 and 107 respectively) [25]. Although 3-year disease-specific survivals (DSS) with RT-CT, AccRT and HypoRT were statistically non-significant (80%, 81% and 74%, respectively, *P* = 0.219), Cox regression analysis identified treatment modality as a significant factor affecting DSS (*P* = 0.038). Compared with RT-CT, the hazard ratio (HR) for DSS was 1.0 with AccRT and 2.0 with HypoRT (*P* = 0.021). Kaplan–Meier comparisons found no significant difference in LRC and OS between RT-CT and AccRT. HypoRT was associated with a significantly lower LRC (*P* = 0.005) and OS (*P* = 0.008) compared with RT-CT. They concluded that in patients with locally advanced oropharyngeal cancer, AccRT conferred DSS, LRC and OS comparable to that of RT-CT. Patients treated with RT-CT experienced higher rates of treatment-related acute toxicities. Hypofractionation had the least favorable outcome. Although the retrospective design limits the impact of Kader *et al*., the treatment outcome and toxicities of both RT–CT and AccRT compare favorably with those of the CCRT and CCB arms in our study.

The latest publication of CCRT vs accelerated radiotherapy with or without concomitant chemotherapy for locally advanced head and neck carcinoma (GORTEC 99-02) [26] reported promising results for CCRT. In this study, 840 patients with locally advanced, Stage III–IV (non-metastatic) SCCHN and an ECOG performance status of 0*–*2 were randomly allocated in a 1:1:1 ratio to receive conventional chemoradiotherapy (70 Gy in seven weeks plus three cycles of four days of concurrent carboplatin-5-FU), accelerated radiotherapy-chemotherapy (70 Gy in six weeks plus two cycles of five days of concurrent carboplatin-5-FU), or very accelerated radiotherapy alone (64.8 Gy [1.8 Gy twice daily] in 3.5 weeks). At the median follow-up of 5.2 years, conventional chemoradiotherapy improved progression-free survival (PFS) compared with very accelerated radiotherapy *(P* = 0.041). However, accelerated RT-CT offered no PFS benefit compared with conventional chemoradiotherapy *(P =* 0.88) or very accelerated radiotherapy *(P =* 0.060). The 3-year PFS was 37.6% (95% CI 32.1*–*43.4) after conventional chemoradiotherapy, 34.1% (28.7*–*39.8) after accelerated RT-CT, and 32.2% (27.0*–*37.9) after very accelerated radiotherapy. More patients in the very accelerated radiotherapy group had RTOG Grade 3*–*4 acute mucosal toxicity (226 of 268 patients, 84%) compared with accelerated RT-CT (205 of 271 patients, 76%) or conventional chemoradiotherapy (180 of 262; 69%, *P* = 0.0001). They concluded that conventional chemoradiotherapy had the most favorable outcomes, suggesting that the acceleration of radiotherapy is probably not beneficial in CCRT schedules. A similarity between GORTEC 99-02 and our current study is they both support CCRT over altered fractionation, with our study showing a significant benefit in OS, rather than DFS as in GORTEC. An intriguing difference between the GORTEC 99-02 study and our current study is the fractionation of the second daily dose, in which we used 1.2 Gy and GORTEC 99-02's accelerated arm continued with a second 1.8-Gy daily dose. Grade 3*–*4 acute mucosal toxicity was quite high in both studies, 67.6% in our CCB group (graded by NCI-CTC version 2.0) vs 84% in GORTEC 99-02 (scored by RTOG grading); in addition, severe Grade 3*–*4 late radiation toxicities were similar between the two studies (range, 2.2*–*8% in our CCB arm, and 1*–*11% in the GORTEC trial).

As stated earlier, our two main findings in support of CCRT are a trend of better LRC *(P* = 0.18) and a marginally significant improved OS *(P* = 0.05). The difference in LRC, although not statistically significant, appeared to have an impact on OS. There might be an association between LRC and OS, but this study was underpowered to detect it. Two caveats regarding the interpretation of OS benefit are the higher median age of patients in the CCB arm vs the CCRT arm (63.0 vs 58.5 years), and the failure to recruit sufficient patients in this small study. A final factor that could have affected the OS could be the lower compliance with radiotherapy seen in the CCRT group.

## CONCLUSION

In conclusion, our results indicated that CCRT produced a trend towards better outcome compared with CCB irradiation. However, the higher hematologic toxicities and poor radiotherapy compliance rate are important limiting factors to be considered regarding this treatment.
